# A transcriptomic signature for prostate cancer relapse prediction identified from the differentially expressed genes between TP53 mutant and wild-type tumors

**DOI:** 10.1038/s41598-022-14436-y

**Published:** 2022-06-22

**Authors:** Wensheng Zhang, Kun Zhang

**Affiliations:** 1 Bioinformatics Core of Xavier NIH RCMI Center of Cancer Research, Xavier University of Louisiana, New Orleans, LA 70125 USA; 2 Department of Computer Science, Xavier University of Louisiana, New Orleans, LA 70125 USA

**Keywords:** Cancer epidemiology, Cancer genetics, Cancer genomics, Cancer prevention, Tumour biomarkers, Urological cancer, Genetics, Biomarkers, Molecular medicine, Oncology

## Abstract

For prostate cancer (PCa) patients, biochemical recurrence (BCR) is the first sign of disease relapse and the subsequent metastasis. *TP53* mutations are relatively prevalent in advanced PCa forms. We aimed to utilize this knowledge to identify robust transcriptomic signatures for BCR prediction in patients with Gleason score ≥ 7 cancers, which cause most PCa deaths. Using the TCGA-PRAD dataset and the novel data-driven stochastic approach proposed in this study, we identified a 25-gene signature from the genes whose expression in tumors was associated with TP53 mutation statuses. The predictive strength of the signature was assessed by AUC and Fisher’s exact test p-value according to the output of support vector machine-based cross validation. For the TCGA-PRAD dataset, the AUC and p-value were 0.837 and 5 × 10^–13^, respectively. For five external datasets, the AUCs and p-values ranged from 0.632 to 0.794 and 6 × 10^–2^ to 5 × 10^–5^, respectively. The signature also performed well in predicting relapse-free survival (RFS). The signature-based transcriptomic risk scores (TRS) explained 28.2% of variation in RFS on average. The combination of TRS and clinicopathologic prognostic factors explained 23–72% of variation in RFS, with a median of 54.5%. Our method and findings are useful for developing new prognostic tools in PCa and other cancers.

## Introduction

The state of an increasing prostate-specific antigen level after radical prostatectomy (RP) or radiation therapy (RT) for localized prostate cancer (PCa) is known as biochemical recurrence (BCR) or biochemical relapse. The rate of BCR following RP was estimated to be 20‑40%. BCR is the first sign for disease relapse and subsequent lethal metastases^1^, occurring within a wide time span from a few months to over 15 years following the initial therapy^2^. Cases of PCa progression with undetectable or low PSA levels have rarely been observed^3,4^. In the absence of secondary treatment, patients with BCR have an approximate median period of 5–8 years prior to clinical progression^2,3^. BCR events usually occur among patients with at least one of the primary and secondary prevalent Gleason patterns (GPs) being graded as 4 or 5. BCR risk and disease-specific mortality increase with the climbing proportions of the GP-4 and GP-5 components, from 3 + 3 with tertiary 4 to 3 + 4, 4 + 3 and 4 + 4, in prostatectomy specimens^5–7^. Prostate cancer without GP-4 or GP-5 components is unable to metastasize or cause cancer-associated mortality, in addition to having a low risk rate for BCR^8,9^. BCR and BCR-free survival are significantly associated with overall survival (OS), but they are poorer as surrogate endpoints for OS than metastasis-free survival^10^.

The Gleason score (GS) is the sum of the grades of the first and second Gleason patterns of a primary cancer sample. Within the same GS group, individual cancers have heterogeneous molecular mechanisms, implicating varied progression potential. In particular, even patients with GS ≥ 8 can experience favorable oncological outcomes^11^. In this regard, stratifying GS ≥ 7 into relapse risk groups is pivotal to the management of prostate cancer. For example, it can be helpful for scheduling follow-up surveillance after the initial treatment.

In past years, several studies have focused on mining predictive marker sets (signatures) for BCR or relapse-free survival (RFS) from high-throughput molecular data resources. The numbers of the involved genes in the identified signatures range from four to a few dozen^12–16^. While those signatures were valid for the datasets or experimental settings in the reported studies, their robustness and prognostic utility could be uncertain due to drawbacks in the design/analysis, such as the inclusion of low-risk GS-6 cancer samples in the datasets used^13,14,16^ and/or the lack of sufficient validation using external datasets^14,16^. Moreover, the strategy of identifying a signature for the prediction of cancer relapse from the top differentially expressed genes between tumors and normal samples or between benign tumors and malignant tumors was usually adopted in those studies^12,14^. As such, overfitting likely arose due to the lack of a mechanism to reduce the risk of its occurrence. An additional challenge is that a molecular signature with a practical application perspective should complement clinicopathologic prognostic factors such as GS in outcome prediction. In a recent report, Wu et al^12^ addressed this issue, but our preliminary analysis demonstrated that their result obtained from analyzing the Cancer Genome Atlas Prostate Adenocarcinoma (TCGA-PRAD) dataset could not be confirmed using other datasets (see Results section).

In this study, we aimed to identify a robust predictive gene expression signature for BCR in patients with Gleason score (GS) ≥ 7 cancers. To achieve our goal, a data-driven and biologically informed stochastic approach was developed, which begins with the use of the TCGA-PRAD dataset to identify differentially expressed genes regarding *TP53* mutation status in cancer samples. In the cohort, *TP53* mutations are less frequent than *PTEN* mutations but are more enriched than aberrations in other key oncogenes and/or tumor suppressors such as *KRAS*, *BRAF*, *EGFR* and *MYC*^17^. Our methodology conceived with the hypotheses that a TP53 mutation status-associated prognostic gene signature could be robust, namely, the prediction strength for BCR could keep when the gene expression levels in the datasets of different patient cohorts are measured with varied platforms and experimental settings. Underlying this perception is the fact or finding that the prevalence of somatic *TP53* mutations in advanced forms of PCa is approximately 4 times the quantity in primary cancer^17,18^, which suggests that the genes with *TP53* mutation status-associated transcription could be enriched with prognostic and etiological factors for cancer relapse.

## Material and methods

### Signature discovery

#### Scheme

**Figure 1:**
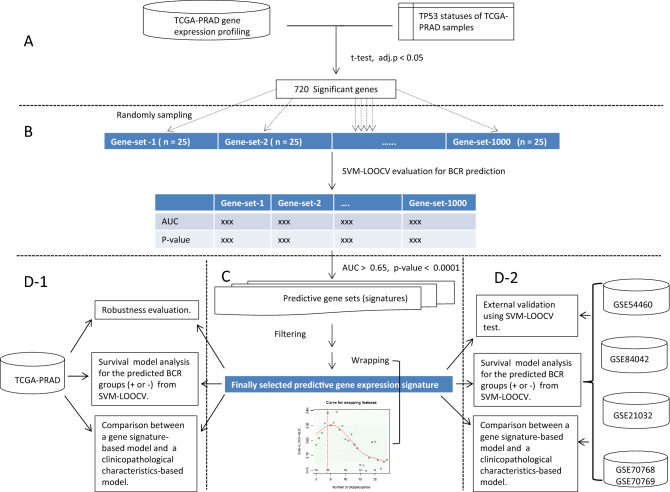
Flow chart of the identification approach (**A,B**,**C**) and performance/utility evaluation (**D-1**, **D-2**) of a *TP53* mutation status-associated predictive transcriptomic signature for BCR. In **A**, the top differentially expressed genes (DEGs) regarding TP53 statuses were identified. In (**B**), 1000 small subsets of the DEGs were randomly sampled and their predictive strengths for BCR were assessed by SVM-based cross validation. In (**C**), the results from (**B**) step were integrated by a “filter” and a novel “wrapper” to obtain an optimized gene signature. In (**D1**) and (**D2**), the performance of the finally selected signature for BCR prediction and the clinical utility were evaluated in the TCGA dataset and five external datasets using statistical and machine learning methods. See the main text for a more detailed explanation.

The stochastic approach we have developed for identifying a gene expression signature for BCR is data-driven and biologically informed. It includes three modules (A, B and C parts of Fig. 1). First, using the TCGA-PRAD dataset, the genes differentially expressed between the tumors with somatic *TP53* mutation(s) and those without mutations on the gene are detected. Second, 1000 small (size = 25. See the “*Remark*” paragraph) subsets of genes are randomly sampled from the output of first modules, and their predictive strengths for BCR are assessed with the area under the receiver operating characteristic curve (AUC) and Fisher’s exact test p-value. For each of the gene subsets (random signatures), the two performance metrics are calculated according to the predicted BCR risk category and decision values of individual subjects from support vector machine-based leave-one-out cross validation (SVM-LOOCV). Third, the results from the second modules are integrated using a “filter” and a “wrapper” to obtain an optimized gene signature.

#### Filter

The filter works via two operations. OP-1: The gene subsets whose AUCs and p-values meet the cutoffs of ≥ 0.65 and ≤ 0.0001 (see the “*Remark*” paragraph) are selected. OP-2: The genes with at least three hits in the selected subsets are collected as the “initial” signature.

#### Wrapper

Suppose the initial signature contains *m* genes and denote it by a character vector ${\mathcal{G}} = \left\{ {g_{1} ,g_{2} \ldots \ldots ,g_{m} } \right\}$. The wrapper is designed to refine ${\mathcal{G}}$ and is realized via the following algorithm.

1. A numeric vector $A = \left\{ {A_{{\left( { - 1} \right)}} ,A_{{\left( { - 2} \right)}} \ldots \ldots A_{{\left( { - m} \right)}} } \right\}$ is calculated, where $A_{{\left( { - i} \right)}}$ represents the SVM-LOOCV AUC obtained when all the genes in ${\mathcal{G}}$ except for $g_{i}$ are used as the features for predicting BCR.
2. The elements of vector *A* are sorted in ascending order to generate $A^{*} = \left\{ {A_{1}^{*} , A_{2}^{*} \ldots \ldots A_{m}^{*} } \right\}$. Correspondingly, the gene vector ${\mathcal{G}}$ is rearranged to generate ${\mathcal{G}}^{*} = \left\{ {g_{1}^{*} ,g_{2}^{*} \ldots \ldots ,g_{m}^{*} } \right\}$.
3. Specify the lower limit of the size of a desired signature, such as 10, and denote it with *k*. A numeric vector $B = \left\{ {B_{{\left( { - 1:1} \right)}} ,B_{{\left( { - 1:2} \right)}} \ldots \ldots B_{{(-1:\left( {m - k} \right))}} } \right\}$ is calculated, where $B_{{\left( { - 1:i} \right)}}$ represents the SVM-LOOCV AUC obtained when the genes $\left\{ {g_{i + 1}^{*} ,g_{i + 2}^{*} \ldots \ldots ,g_{m}^{*} } \right\}$ are used as the prediction features. The genes corresponding to individual elements of *B* are counted, and the numbers are collected into an integer vector *C*.
4. Create scatter plot with *C* as x-axis and *B* as y-axis, and model the relationship between C and B with a single-mode smooth-splining curve. The data point adjacent to the mode of the curve from the left side is visually pinpointed, and the corresponding gene set (a subset of ${\mathcal{G}}^{*}$) is determined as the finally selected signature.

#### Remark

The parameters used in the discovery procedure were set by referring to the results of a preliminary study. These results include (1) a random signature consisting of 10–200 genes rarely demonstrated a prediction strength of AUC ≥ 0.70 and p ≤ 0.00001; and (2) among numbers 10, 25, 50, 100 and 200, the second was the best as the size of the gene subset for efficiently selecting candidate prognostic signatures that could meet the modestly specified performance criteria.

After the signature for BCR prediction was identified, its robustness and prognostic utility were future evaluated by the analyses outlined in the parts D1 and D2 of the Fig. 1.

### Statistics and machine learning methods

#### SVM-LOOCV

First, based on the clinical outcomes regarding binary BCR occurrence, the N cancer cases in a cohort are divided into two classes: BCR^-^ (“−1” group) and BCR^+^ (“1” group). The labels of these cancer cases are then saved in a vector $Y = (y_{1} , y_{2} , \ldots y_{i} , \ldots y_{N}$), where $y_{i} \in \left( { - 1,1} \right)$. After that, the (assumedly unknown) class of a leave-out tumor *i* is predicted from its gene expression profiling ($\mathop{x}\limits^{\rightharpoonup} _{i} )$ of the signature genes by the SVM model, which is trained on the data ({$\mathop{x}\limits^{\rightharpoonup} _{j} ; y_{j} \}$) of the other N−1 samples. That is,

$$\hat{z}_{i} = sign\left( {t_{i} } \right), t_{i} = \mathop \sum \limits_{j \in S}^{j \ne i} a_{j} y_{j} k\left( {\mathop{x}\limits^{\rightharpoonup} _{j} , \mathop{x}\limits^{\rightharpoonup} _{i} } \right) + b, S = \left\{ {1, 2, 3, \ldots \ldots , N} \right\}$$

In the equations, $\hat{z}_{i}$ denotes the predicted category (1 or −1) for the *i*th sample; $t_{i}$ is the decision value, $k\left( {\mathop{x}\limits^{\rightharpoonup} _{j} , \mathop{x}\limits^{\rightharpoonup} _{i} } \right)$ is the kernel function, and $\{ a_{j}$} and *b* are the model parameters decided in the previous training process. Third, by summarizing the true label vector Y and the output label (i.e., predicted label) vector $\hat{Z} = \left( {\hat{z}_{1} , \hat{z}_{2} , \hat{z}_{3} , \ldots \ldots \hat{z}_{N} } \right)$, a $2 \times 2$ contingency table is generated, on which Fisher’s test of independence is performed. Finally, by combining the true label vector Y and an assemblage of tumor sample classifications based on the vector of decision values $T=\left( {t_{1} , t_{2} , t_{3} , \ldots \ldots t_{N} } \right)$, i.e. the transcriptomic risk scores estimated from SVM-LOOCV, and serially changed cutoffs, a receiver operating characteristic curve is generated and the AUC is calculated.

#### Survival analysis

The association between the predicted BCR groups and relapse-free survival (RFS) was evaluated with the p-value from the log-rank test. The performance comparison between transcriptomic risk scores and clinicopathologic prognostic factors was conducted using Cox-PH regression models. The explained variation (R^2^), i.e., the proportion of variability in the outcome variable RFS explained by the explanatory variable(s), was calculated using Royston’s method^19^. The goodness-of-fit of a survival model was also evaluated with Schwarz's Bayesian information criterion (BIC). When picking from several models, the model with a lower BIC value is generally preferred.

#### Software and application notes

Statistical/computational analysis was completed using the relevant functions in the R packages “stats”, “01,071”, “AUC”, “survival” and “survMisc”, as well as our labor-owned R codes. In the implementation of the *SVM()* function, except for the specially noted cases, a linear kernel was used, the class weights were specified as the reciprocals of the ratios between the “1” samples to the “−1” samples in the training set, and defaults for the hyperparameter *cost* and *gamma* were held on. The p-value from two-tailed Fisher’s exact test was calculated in evaluating the randomly sampled signatures (gene subsets), and the p-value from one-tailed Fisher’s exact test was calculated in evaluating the finally identified signature. In the analyses where BCR was treated as a binary endpoint, the time from the initial PCa diagnosis to relapse for a BCR^+^ sample and to the end of follow-up for a BCR^-^ sample were not considered.

### Data

The clinical data of the six cohorts used as the discovery set (the TCGA-PRAD cohort) or testing/validation cohorts (the GSE54460 and others) in this study are summarized in Table 1. While these cohorts were filtered by the criterion of GS ≥ 7 preceding our advanced analysis, we still denoted them with the IDs given in the source database. The following is a brief description of the gene expression datasets of those cohorts.

**Table 1:** Summary of the used datasets regarding clinicopathologic characteristics of patients.

Data ID	Sample sizes	Sample partition on Gleason pattern (primary + second)			Sample partition on clinical T-stage				BCR %	Interquartile of ages at diag	
		3 + 4	4 + 3	3 + 5, 4 + 4|5, 5 + 4|5	T1	T2	T3	NA		Q2	Q3
TCGA-PRAD	366	114	83	169	124	133	48	61	13.6	57.0	66.5
GSE54460	95	56	24	15	10	67	18	0	53.7	56.7	66.3
GSE84042	57	40	17	0	0	28	29	0	24.6	56.2	63.9
GSE21032	89	53	21	15	43	42	4	0	28.1	54.3	61.8
GSE70768	95	65	21	9	47	32	14	2	20.0	56.0	65.0
GSE70769	70	36	19	15	26	32	9	3	62.9	NA	NA

#### Discovery dataset

The level-3 gene expression dataset (version 2) of TCGA-PRAD samples was downloaded from the Genomic Data Commons Data Portal. The TCGA group performed RNA-Seq experiments on an Illumina HiSeq platform and estimated the gene expression levels by transcripts per million (TPM) values using an expectation maximization method and RSEM software^20^. Log2 transformation on this dataset was performed preceding the analysis.

#### Testing (validation) datasets

All the gene expression datasets (the series matrices) of the testing cohorts were downloaded from the Gene Expression Omnibus (GEO) database. The authors of GSE54460^21^ performed RNA-Seq experiments with the Illumina HiSeq 2000 platform, mapped shorts reads on the human genome hg19 assembly using TopHat and Bowtie software, and estimated gene expression levels with fragments per kilobase million (FPKM) values using Cufflinks software. The authors of GSE84042^22^ performed microarray experiments with Affymetrix Human Gene 2.0 ST array and preprocessed expression intensities using the robust multichip average (RMA) algorithm^23^ and log2 transformation. The authors of GSE21032^24^ performed microarray experiments using Affymetrix Human Exon 1.0 ST Array and preprocessed expression intensities using RMA and quantile normalization. We performed log2 transformation on the downloaded dataset, which contained transcript (Refseq RNA) expression levels. For a gene with two or multiple transcript IDs, we chose the one whose expression levels across samples had the largest interquartile range (IRQ) as the representative. The raw data of GSE70768 and GSE70769 were generated by the same authors using Illumina HumanHT-12 V4.0 Expression BeadChip^16^. We first downloaded the two matrices of the non-normalized expression levels of the two cohorts and removed the columns for the samples that would not be used in our analysis. Then, quantile normalization and log2 transformation were applied. For a gene with two or multiple probes, we chose the one whose expression levels across samples had the largest IRQ in the GSE70768 cohort as the representative. Finally, we homogenized the two normalized expression matrices to make them have the same global 75% quantile.

## Result

### *TP53* mutation status-associated (*TP53*-mut-ass) genes

We selected 720 *TP53*-mut-ass genes via the following procedure. First, based on the entire expression matrix of the TCGA-PRAD cohort, the genes unexpressed in at least half the samples were filtered out. The expression levels of those excluded genes were typically very low, even in the samples where the quantities were not zero. As such, we assume they may be not activated in prostate tissue actually and could hardly save as reliable prognostic features. Second, a t-test for the difference in the average expression levels between *TP53*-mutant and wild-type GS ≥ 7 samples was performed to scan the ~ 17,700 genes that remained after the first step. Third, the p-values estimated in the second step were adjusted by the BH method and adj. p < 0.05 was used as the cutoff for gene selection.

### Transcriptomic signature for BCR

We identified a 25-gene transcriptomic signature from the *TP53*-mut-ass genes for predicting BCR. Coincidentally, the number of member genes was equal to the size of a random signature tested in the second module of our approach. It was also within the range of sizes of PCa prognostic gene signatures identified by other research groups^12–16^. The signature was not enriched with the genes in any gene ontology (GO) term or KEGG pathway, as demonstrated by a functional enrichment analysis using the DAVID tool/database^50^. However, it was enriched (p = 0.02, from Fisher’s exact test) with the cancer gene census (downloaded on April 16, 2020) established by the Catalog of Somatic Mutations in Cancer (https://cancer.sanger.ac.uk/cosmic). The cancer genes contained in the census included *CDKN1A*, *LARP4B* and *SLC45A3*. Previous studies showed that *LARP4*B inhibited the migration and invasion of prostate cancer cells^32^, *SLC45A3* downregulation was significantly associated with shorter PSA-free survival times, and the expression of *SLC45A3* protein was downregulated through *SLC45A3-ERG* fusion^51^. Moreover, a recent study demonstrated that another 16 genes in the signature were relevant to the formation and progression of prostate cancer and/or other cancers (Table 2). For example, Zhao et al. found that *SMC4* knockdown reduced migration and/or invasion of cancer cells and that outlier expression of the gene was significantly associated with poor PCa prognosis^45^, and Jiang et al. showed that overexpression of *SMC4* activated *TGFβ/Smad* signaling and promoted an aggressive phenotype in glioma cells^44^.

**Table 2:** Signature genes and their relevance to PCa and/or other cancers.

Symbol	Name	Relevance with cancer/tumor/patient and references
*CDKN1A*	cyclin dependent kinase inhibitor 1A	Variants; advanced PCa^25^
*DDB1*	damage specific DNA binding protein 1	Apoptosis, chemo-resistance regulation and progression; multiple cancer types^26–28^
*EIF5A2*	eukaryotic translation initiation factor 5A2	Cell growth, metastasis, chemotherapy resistance; multiple cancer types^29,30^
*GCDH*	glutaryl-CoA dehydrogenase	
*GK3P*	glycerol kinase 3 pseudogene	
KIAA0196	Strumpellin	Amplified and overexpressed; PCa^31^
*LARP4B*	La ribonucleoprotein 4B	Cell migration and invasion; PCa^32^
*NAA50 (NAT13)*	N-alpha-acetyltransferase 50, NatE catalytic subunit	
*NDUFA9*	NADH: ubiquinone oxidoreductase subunit A9	Cell Proliferation, Metastasis; breast cancer^33^
*NFATC3*	nuclear factor of activated T cells 3	Tumor growth, cell proliferation and migration; astroglioma^34^
*NUMB*	NUMB endocytic adaptor protein	Invasion, metastasis, migration; melanoma ^35^, colon cancer^36^
*OIP5*	Opa interacting protein 5	Growth, metastasis and drug-resistance; bladder cancer^37^
*PFKFB2*	6-phosphofructo-2-kinase/fructose-2,6-biphosphatase 2	Glycolysis, cell proliferation; pancreatic cancer^38^
PLEKHF2	pleckstrin homology and FYVE domain containing 2	Amplified, Survival; PCa^39^
*RASIP1*	Ras interacting protein 1	Cell migration; non-small-cell lung cancer cells lines^40^
*RNF167*	ring finger protein 167	Activates mTORC1 and promotes tumorigenesis; breast and liver cancer cell lines. ^41^
SELENBP1	selenium binding protein 1	Tumor growth, progression, survival; lung cancer^42^, PCa^43^
*SLC45A3*	solute carrier family 45 member 3	SLC45A3-ERG fusion, survival; PCa^43^
SMC4	structural maintenance of chromosomes 4	TGFβ/Smad signaling, cell invasion; glioma cells^44^, PCa^45^
*TMEM87A*	transmembrane protein 87A	Cell proliferation and metastasis; gastric cancer^46^
*UBXN2B*	UBX domain protein 2B	
*SRSF10 (SFRS13A)*	serine and arginine rich splicing factor 10	Maintenance of oncogenic features; colon cancer cells^47^
C3orf67	Chromosome 3 open reading frame 67	
C14orf169 (NO66)	Chromosome 14 open reading frame 169	Osteolytic lesions, invasion and metastasis; PCa^48^
*LOC678655 (CD27-AS1)*	CD27 antisense RNA 1	Progression; acute myeloid leukemia^49^

### Predicting BCR

**Figure 2:**
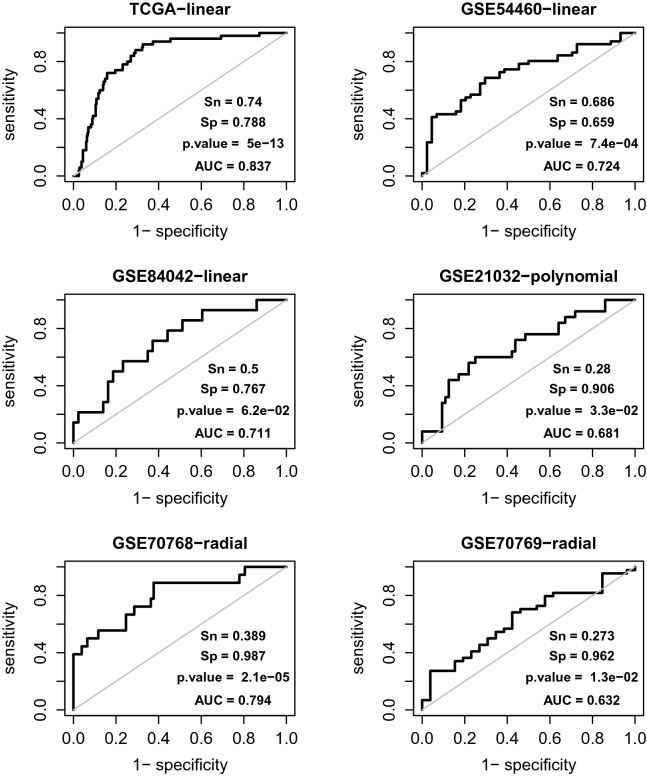
The performance of the TP53 mutation status-associated transcriptomic signature for BCR prediction in the discovery dataset TCGA (-PRAD) and five external datasets, i.e., the GSE54460 and others. The “-linear”, “-polynomial” and “-radial” indicate the kernel functions used in the SVM models. The output BCR label and decision value, i.e. the transcriptomic risk score (TRS) of a patient in GSE70769 was predicted by the model trained using the GSE70768 dataset. For the patients in other cohorts, the labels and scores were predicted via LOOCV. Together with the actual BCR labels, the output BCR labels and TRSs are used to calculate a 2 × 2 contingency table for estimating the p-value and to generate the ROC curve, respectively. Sn and Sp denote sensitivity and specificity, respectively.

The performance of the identified signature for BCR prediction was finally evaluated using the TCGA-PRAD dataset and validated using five external datasets (Fig. 2**).**

While SVM-LOOCV, a strategy appropriate for accurately estimating the performance of a classification model, was adopted, testing on the TCGA-PRAD cohort was subject to overfitting. Namely, the obtained prediction strength (AUC = 0.837; p-value = $5 \times 10^{ - 13}$) could be overestimated for the patient population(s) represented by this cohort. The reason was that the gene signature was discovered in the same dataset. In this regard, we performed a complementary analysis to verify the prediction robustness, i.e., to demonstrate that the observed performance was not due to the specific data body exactly consisting of the expression profiling and BCR statuses of all 366 focused samples. Briefly, 500 working sets with size n = 330 (366 × 0.9) were generated from the TCGA-PRAD dataset by non-replacement sampling, and on each of them, the SVM-LOOCV AUC was estimated after its expression matrix was altered by artificial noise. The noises were introduced by a two-step procedure: (i) the expression levels of the signature genes were rescaled by z-transformation, and (ii) random noises $x\sim norm\left( {0, 0.1} \right)$ were added to the standardized expression metrics (in this setting, the variance of the introduced noise was equal to 10% data variance). The results from this analysis showed that the 0.05 and 0.95 quantiles of the 500 AUC values were 0.688 and 0.803, respectively, and only 0.6% of them were less than 0.65.

The results from GSE54460 and GSE84042 clearly validated the signature. First, both of the two SVM-LOOCV AUC values were over 0.70. Second, in establishing the SVM model, a simple linear kernel was used, and no hyperparameters were tuned. For the GSE21032 dataset, the output from the polynomial-kernel (with defaults for other hyperparameters) SVM model was somewhat better than that from the linear-kernel model (0.681 vs 0.652). For the GSE70768 and GSE70769 datasets, tuned hyperparameters (i.e., *cost* and *gamma*) were required to obtain prediction strength. In such a scenario, the final performance had to be assessed on an independent dataset. Due to the limited cohort sizes and the imbalance in BCR statuses, we could not partition either of the GSE70768 and GSE70769 datasets into a substantial training subset and a substantial testing subset. As such, we addressed these two datasets by considering the former as the training set and the latter as the testing set, the same setting used in the original study^16^. With the optimized *cost* and *gamma* (0.105 and two-thirds the inverse of the feature dimension), the training set had an SVM-LOOCV AUC of 0.794 and a p-value of $2.1 \times 10^{ - 5}$. When the model trained with all the samples in the training set was used to predict the samples in the testing set, the external validation AUC and p-value were 0.632 and $1.3 \times 10^{ - 2}$, respectively.

### Predicting RFS

**Figure 3:**
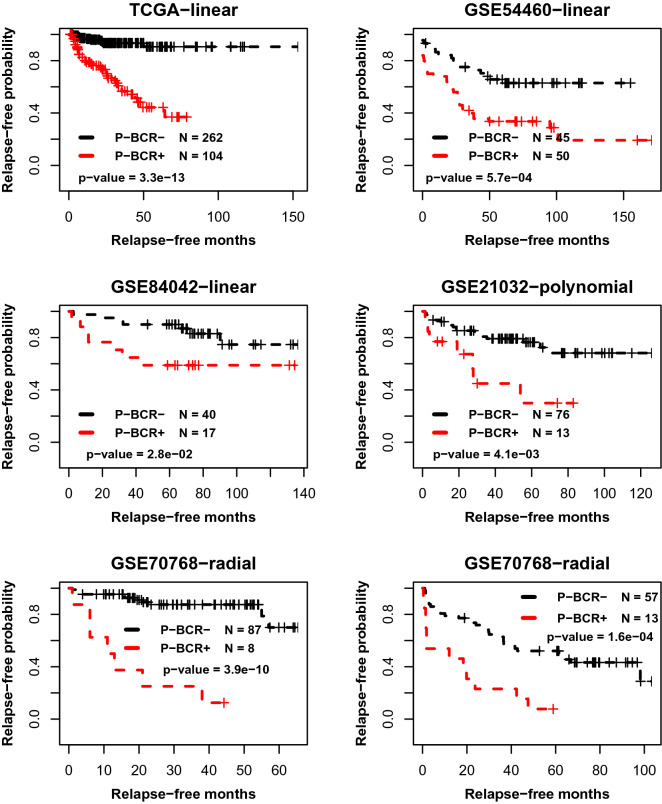
The association between RFS stratification and the BCR partition predicted using the TP53 mutation status-associated prognostic transcriptomic signature in the discovery dataset TCGA (-PRAD) and five external datasets, i.e., the GSE54460 and others. The “-linear”, “-polynomial” and “-radial” indicate the kernel functions in used the SVM models. The output BCR label (pre-BCR^+^ and pre-BCR^-^) of a patient in GSE70769 is predicted by the model trained using the GSE70768 dataset. For the patients in other cohorts, the labels are predicted via LOOCV. The survival profiles of pre-BCR^+^ and pre-BCR^-^ samples are depicted by red and black Kaplan–Meier curves, respectively.

We further evaluated the prognostic performance of the identified signature by testing the association between the stratification of disease relapse-free survival and the predicted BCR partition via SVM-LOOCV. Suppose pre-BCR^+^ and pre-BCR^-^ represent the predicted “positive” and “negative” groups, respectively. The results of survival analysis showed that for each cohort, the Kaplan–Meier curve of the pre-BCR^-^ group was better than the curve of pre-BCR^+^ (Fig. 3). The p-values from the log-rank test of the differences ranged from $2.8 \times 10^{ - 2}$ (for the GSE84042 cohort) to $3.3 \times 10^{ - 13}$ (for the TCGA-PRAD cohort).

### Practical utility: compared with clinicopathologic factors

We deciphered the practical utility of the identified gene signature by comparing its capability to stratify patient RFS with that of clinicopathologic prognostic factors (CPFs). The considered CPFs consisted of Gleason patterns (GPs), T stages (T1, T2, T3) and patient ages at initial PCa diagnosis. In particular, regarding GPs, we partitioned patients into three categories, i.e., 3 + 4, 4 + 3 and the other. On each dataset (cohort), the analysis was conducted by running three Cox-PH regression models. The first (M-1) included the transcriptomic risk score (TRS), which was estimated by the decision value outputted from SVM-LOOCV for individual subjects, as the only explanatory variable. The second (M-2) and third (M-3) included the three CPFs or both the TRS and CPFs as the explanatory variables, respectively. The performance and validity of a model were assessed by the explained variation (R^2^), BIC and global log rank p-value.

Except for M2 in GSE84042, all three models were significant (p < 0.02) in the six cohorts. M-1 performed better than M-2 in GSE54460, GSE84042 and GSE70768 in terms of the higher R^2^ values and/or lower BIC values but was poorer in the other three cohorts. All the R^2^ values from M-3 were higher than those from both M-1 and M-2, while its BIC value in GSE21032 was slightly higher than the score of M-2. From the statistics itemized in Table 3, we derived two conclusions about the prognostic utility of our gene signature. First, TRS could explain 9–60% of the variation in RFS, with an average of 28.2%. Second, TRS could replace and/or complement CPFs to predict RFS, and the combination of TRS and CPFs could explain 23–72% of the variation in RFS, with a median of 54.5%.

**Table 3:** Results from Cox regression model analysis.^╫^

Data ID	M-1, TRS			M-2, CPFs			M-3, TRS + CPFs		
	R^2^	BIC^¶^	p-value	R^2^	BIC	p-value	R^2^	BIC	p-value
TCGA-PRAD	0.43	475.18	1.1 × 10^–10^	0.44	421.18	5.8 × 10^–7^	0.63	403.45	2.2 × 10^–11^
GSE54460	0.12	417.59	5.8 × 10^–4^	0.16	429.32	7.5 × 10^–3^	0.23	426.79	7.6 × 10^–4^
GSE84042	0.36	99.29	2.2 × 10^–3^	0.17	109.63	2.6 × 10^–1^	0.51	104.67	1.3 × 10^–2^
GSE21032	0.09	205.6	7.4 × 10^–3^	0.58	192.68	8.5 × 10^–9^	0.58	195.52	2.1 × 10^–8^
GSE70768	0.60	126.27	9.2 × 10^–8^	0.41	143.26	1.9 × 10^–3^	0.72	130.37	9.8 × 10^–8^
GSE70769	0.09	327.67	9.0 × 10^–3^	0.17	308.64	9.9 × 10^–3^	0.25	306.2	1.2 × 10^–3^

^╫^The three models (M-1, M-2 and M-3) are specified by the included predictor variable(s) for cancer relapse-free survival. TRS: transcriptomic risk score. CPFs: clinicopathologic prognostic factors. See the main text for a more detailed description.

^¶^BIC: Bayesian Information Criterion.

### Reevaluating the documented signatures

To demonstrate the relative advantage of our signature, we reevaluated the prognostic performance of five transcriptomic signatures identified by other researchers. Here, we provide a brief description of those signatures before discussing the results. The methods used in the identification are outlined in Supplementary Text 1.

#### Wu’s signature

The 10-gene signature was identified for predicting BCR in the GS ≥ 7 patient set^12^. The dataset of 414 TCGA-PRAD prostate adenocarcinoma samples (including 37 GS = 6 samples) was used as the discovery (training) set.

#### Li’s signature

The signature consisted of 74 gene pairs from a combination of 60 genes^13^. It was identified to predict BCR, regardless of Gleason scores or patterns. The entire GSE21032 (N = 131) cohort was used as the discovery set. The 60 genes were considered individual features in our analysis. Li et al.’s work also included the analysis of two datasets (i.e., GSE46602 and GSE40272) that were not addressed in our study. The reasons were that there were only 20 GS ≥ 7 samples in the former, and Gleason score information is unavailable in the latter.

#### Komisarof’s signature

The signature included four cooperation response (to oncogenic mutations) genes^14,52^. It was identified to predict BCR, regardless of Gleason scores or patterns. The discovery cohort consisted of 32 samples.

#### Erho’s signature

The signature consisted of 22 features (genome fragments) located on the coding or noncoding regions of 19 genes^15^. It was identified to predict early prostate cancer metastasis and is being used in the commercial Decipher Prostate Genomic Test (https://decipherbio.com/). The discovery cohorts contained 359 samples.

#### Knezevic-Klein’s signature

The 17-gene signature was identified to predicts clinical recurrence, prostate cancer death, and adverse pathology^53,54^, and is being used in the commercial Oncotype DX Genomic Prostate Score Test (https://www.oncotypeiq.com/en-US). The discovery cohort consisted of 441 patients.

#### Performance summary

Among these five signatures, only Knezevic-Klein’s signature demonstrated prediction strength for BCR on all the six datasets in the SVM-LOOCV reevaluation (Supplementary Fig. S1, S2, S3, S4, S5). In particular, the BCR statuses of the samples in GSE84042 could not be predicted by any one of the other four signatures. However, Komisarof’s signature in GSE54460 and Li’s signature in GSE21032 had higher AUCs (0.766 vs 0.724 and 0.859 vs 0.681) and lower p-values (1.2 × 10^–4^ vs 7.4 × 10^–4^ and 1.2 × 10^–6^ vs 3.3 × 10^–2^) than our signature, respectively. Here, we specifically noted the following three points. First, due to the preceding information leak in the identification process, the good performance of Li’s signature in GSE21032 might be overestimated. Second, when Li’s signature was applied to the GSE21032 dataset, the polynomial-kernel SVM worked better than the linear-kernel SVM, similar to the situation in evaluating our signature. Third, the modest significant performance (AUC = 0.627 or 0.644, and p < 0.1) of Wu’s and Erho’s signatures on the GSE70768 dataset was obtained after the post hoc optimization of the hyperparameter *cost* and *gamma* of the SVM model, again similar to the situation in evaluating our signature. The last two points indirectly verified the reliability of the observed performance of our signature on these two datasets.

### Extended analysis

In this subsection, we demonstrated that the approach proposed in this study was also efficient for identifying prognostic signatures from the gene set defined by a specific cancer progression-related biological theme. The activity of genes involved in immunology pathways is such a theme^55–57^.

We identified an immune signature of 16 genes (*SPARC, IFNAR2, FOXQ1, G3BP1, IKBIP, BAT1, AZIN1, ZDHHC17, RRAS, DOK7, DMRTA1, ACTG1, AGFG1, M6PR, MED7,* and *PSAPL1*) from ~ 1200 immune function- or regulation-related genes^58^. A couple of minor modifications were made in the method implementation: (i) the number of random signatures to be tested was increased from 1000 to 2000 and (ii) a gene to be selected to the initial signature had to be included in 2 (rather than 3) random signatures that met the criteria of AUC (> 0.65) and p-value (< 0.0001) in predicting the BCR statuses of the TCGA-PRAD samples.

This immune signature was evaluated on the six datasets by the same methods and model settings as used in the aforementioned tests. The results showed that the expression profiles of the signature genes could predict both BCR and RFS (Supplementary Fig. S6, S7). Except for GSE84042, in which it lacked prediction strength, its performance was comparable with the signature extracted from the *TP53-*mut-ass genes.

## Discussion

In this study, using the data generated by the TCGA-PRAD group, we identified a transcriptomic signature to predict BCR in patients with Gleason score ≥ 7 prostate cancer according to gene expression levels measured from prostatectomy specimens. The 25-gene signature was a small portion of 720 genes that were differentially expressed (FDR < 0.05) between the samples with somatic *TP53* mutation(s) and those without *TP53* mutation. However, the prognostic signature is not tied to the mutation statuses and/or expression levels of TP53 itself and TP53 mutation analysis is not a pre-requisite for the expected utilization. The signature was evaluated on the discovery dataset and five external datasets, demonstrating robust prognostic performance not only for predicting BCR but also for stratifying RFS. The risk scores derived from the signature by SVM-LOOCV explained 9–63% of variation in RFS and could complement clinicopathologic prognostic factors.

The advantage of our TP53 mutation status-associated signature was clearly shown in the comparison with those (i.e., Li’s, Wu’s, Komisarof’s, Erho’s and Knezevic-Klein’s) presented in recent literature and/or used in commercial prognostic tools regarding their performance on the same datasets. While Knezevic-Klein’s signature could predict BCR in all the six datasets, its performance was somewhat poorer than our signature in terms of AUCs and p-values, in general. Nevertheless, the reevaluation results of the five external signatures should be carefully scrutinized. For example, because Erho’s signature was identified to predict metastasis, which is preceded by BCR but is not equivalent to BCR, its deficient performance in predicting BCR should not alleviate the potential prognostic utility. Moreover, in the original study, Li’s signature was identified and assessed by their paired-gene expression-based tree model, which might partially explain the poor robustness when it was tested using SVM-LOOCV.

The member genes of our signature were complementary to each other in predicting BCR, although no enrichment relationship was found between them (as a while) and any cancer pathway. Previous studies reported that the activity (expression) of 80% of these genes was related to cancer cell invasion, cancer progression and/or patient outcomes (Table 2). However, according to our additional analysis with univariate regression models, none of them alone could consistently predict BCR and/or RFS in all the six datasets. Even the combination of the five genes, including *LARP4B*, *PLEKHF2*, *SMC4*, *SLC45A3* and *NO66*^32,39,44,45,48,51^, whose clinical relevance and prognostic implications were observed in prostate cancer, had very limited prediction strength for BCR and RFS (not reported in the Results section). In this regard, we perceived that due to the complicated clinical, genetic, pathological and demographical heterogeneity of prostate cancer, a patient cohort may have specific gene expression prognostic factors, and a robust transcriptomic signature should include the genes that could cover such heterogeneity.

Our signature was identified using the data-driven and biologically informed stochastic approach developed in this study. It was characterized with a couple of points. First, a biological information source (i.e., somatic *TP53* mutation profile) critical to cancer progression was used to select a set of candidate signature genes. Naturally, this was a step of introducing external information (knowledge), which has been adopted in our and others’ published studies^59,60^. Second, the ranks of candidate genes regarding their differences in expression levels between BCR-positive and BCR-negative samples were not considered in any steps. Alternatively, the chance of a gene being selected into the initial (and final) signature was determined by the post hoc performance of the random signatures that contained it. As such, the member genes of the final signature did not exclusively consist of the discovery set-specific top BCR-distinguishing genes. Our expectation that such a design could reduce the risk of overfitting was verified by the aforementioned results.

Another novel component of our approach is the algorithm denoted by “wrapper”, which was designed to filter out the genes that erode the prediction strength that can be expected from a reduced signature without them. The wrapper somewhat resembles the backward variable selection (BVS) procedure usually adopted by a multivariable regression analysis, in which the initially selected features (explanatory variables) are ranked based on their statistical significance level for explaining the outcome variable and the feature-dropping process begins from the least significant one. Meanwhile, it conceptually differentiates from BVS in that the benefit from dropping a potentially redundant feature is assessed by the AUC gain outputted from SVM-LOOCV rather than the R^2^ (or its variants) and –log10(p-value) gain from fitting a reduced (linear) model. Therefore, the wrapper actually combines the model optimization of a regression analysis and the model validation step that is highly desired for avoiding overfitting. In this aspect, the wrapper is more similar to the recursive feature elimination (RFE) algorithm proposed in Ref^61^. However, in RFE, redundant features are iteratively dropped due to their relatively minor contribution to the SVM classifier rather than their negative impact on the expected prediction strength.

In the “Extended analysis” subsection of the Results section, we identified an alternative prognostic signature via a modified implementation of our approach, which consisted of 16 immune-related genes. By this, we demonstrated that the approach was also efficient for extracting prognostic signatures from a gene set under a specific cancer progression-related biological theme. Here, we further note that it can be adapted as a general feature selection method, contributing to more general applications of high-throughput data such as the molecular prediction of the subtypes and progression stages of a disease. The adaptation can be worked out by replacing the *TP53*-mut-ass gene set, or immune gene set, with any one that is enriched with the molecular diagnostic/prognostic factors regarding the focused clinical trait in a specific study.

## Supplementary Information

Supplementary Information.

## Data Availability

The used TCGA and GEO datasets reside at https://portal.gdc.cancer.gov/ and https://www.ncbi.nlm.nih.gov/geo/, respectively.
