# Supplementary material for: A transcriptomic signature for prostate cancer relapse prediction identified from the differentially expressed genes between TP53 mutant and wild-type tumors
Source: Sci Rep. 2022 Jun 22;12:10561. doi: 10.1038/s41598-022-14436-y (PMC9217948; doi:10.1038/s41598-022-14436-y)
Supplement: Supplementary file 1 — Supplementary Information. [file 41598_2022_14436_MOESM1_ESM.pdf]

Supplementary Information for "A transcriptomic signature for prostate cancer relapse prediction identified from the differentially expressed genes between TP53 mutant and wild-type tumors"

Wensheng Zhang[1], Kun Zhang[1,2]

[1]Bioinformatics Core of Xavier NIH RCMI Center of Cancer Research, Xavier University of Louisiana, New Orleans, LA 70125, USA

[2] Department of Computer Science, Xavier University of Louisiana, New Orleans, LA 70125, USA

Content

Supplementary Text 1, page 2

Supplementary Fig. S1, page 3

Supplementary Fig. S2, page 4

Supplementary Fig. S3, page 5

Supplementary Fig. S4, page 6

Supplementary Fig. S5, page 7

Supplementary Fig. S6, page 8

Supplementary Fig. S7, page 9

Reference, page 10

Supplementary Text 1: Description of external transcriptomic signature

Wu's signature*[1]*: The 10-genes signature was identified to BCR in patients with GS≥7 cancer. The authors used the dataset of 414 TCGA-PRAD samples as the discovery dataset. They firstly selected ~1300 differentially expressed genes (DEGs) between GS=6 and GS≥7 samples using the R package "edgeR". Then, from the DEGs, they identified 39 prognostics genes using the LASSO (least absolute shrinkage and selection operator), Cox-PH regression and 10-fold validation. Finally, the set of prognostics genes was further refined by a multivariate Cox analysis, resulting in the reported signature.

Li's signature*[2]*: The signature consisted of 74 gene pairs of 60 genes. It was identified to predict BCR in PCa patients, regardless of Gleason patterns. The entire GSE21032 cohort, containing 131 samples, was used as the discovery set. With relapse free survival (RFS) as response variable, the authors firstly identified 80 top prognostic genes using the Cox-PH model. Then, from the ~3200 pairs of those genes, they selected 1205 RFS-associated gene pairs. Finally, 74 most significant prognostic gene paired were pinpointed using a forward selection procedure.

Komisarof's signature*[3]*: The signature was identified from cooperation response genes (CRGs), which were synergistically dysregulated in response to cooperating oncogenic mutations[4]. The discovery cohort contained 32 PCa samples, whose gene expression intensity was quantified via Taq-Man Low Density Array RT-PCR. The authors scanned the 95 CRGs to estimate the t-test p-values for the differences between the patients who experienced BCR and those without BCR. The combined performance of top significant genes in predicting BCR was evaluated using three classification algorithms. When the cutoff of p-value for defining top significant genes was set at 0.01, the selected signature of 4 top genes showed the best performance.

Erho's signature*[5]*: The signature consisted of 22 genome fragments (features), located on the coding or noncoding regions of 19 genes. It was identified to predict early metastasis following RP. The discovery cohorts contained 359 samples. Each feature corresponded to a probe set on Human Exon 1.0 ST GeneChips. The authors firstly selected 18,902 differentially expressed features between cases (with metastasis) and controls (without metastasis) using t-test. Then, the initially selected feature set was reduced to a smaller one of 43 features using the regularized logistic regression method. Finally, the 43 features were further filtered to only those that improved a random forest-based performance metric.

Knezevic-Klein's signature [6,7]: The 17-gene signature was identified to predict clinical recurrence, prostate cancer death, and adverse pathology. The discovery cohort consisted of 441 patients. Initially, 732 candidate genes were selected through a meta-analysis of several public microarray data sets (GSE3933, GSE10645, GSE5132 and GSE3325), in which gene expression levels were measured using multiple platforms, including Affymetrix Human Genome U133 Plus 2.0 Array and others. The list of the candidate genes was refined by comprehensive bioinformatics approaches using the data of the discovery cohort, in which the gene expression levels of the prostatectomy samples were measured by TaqMan quantitative reverse transcription–polymerase chain reaction assays.

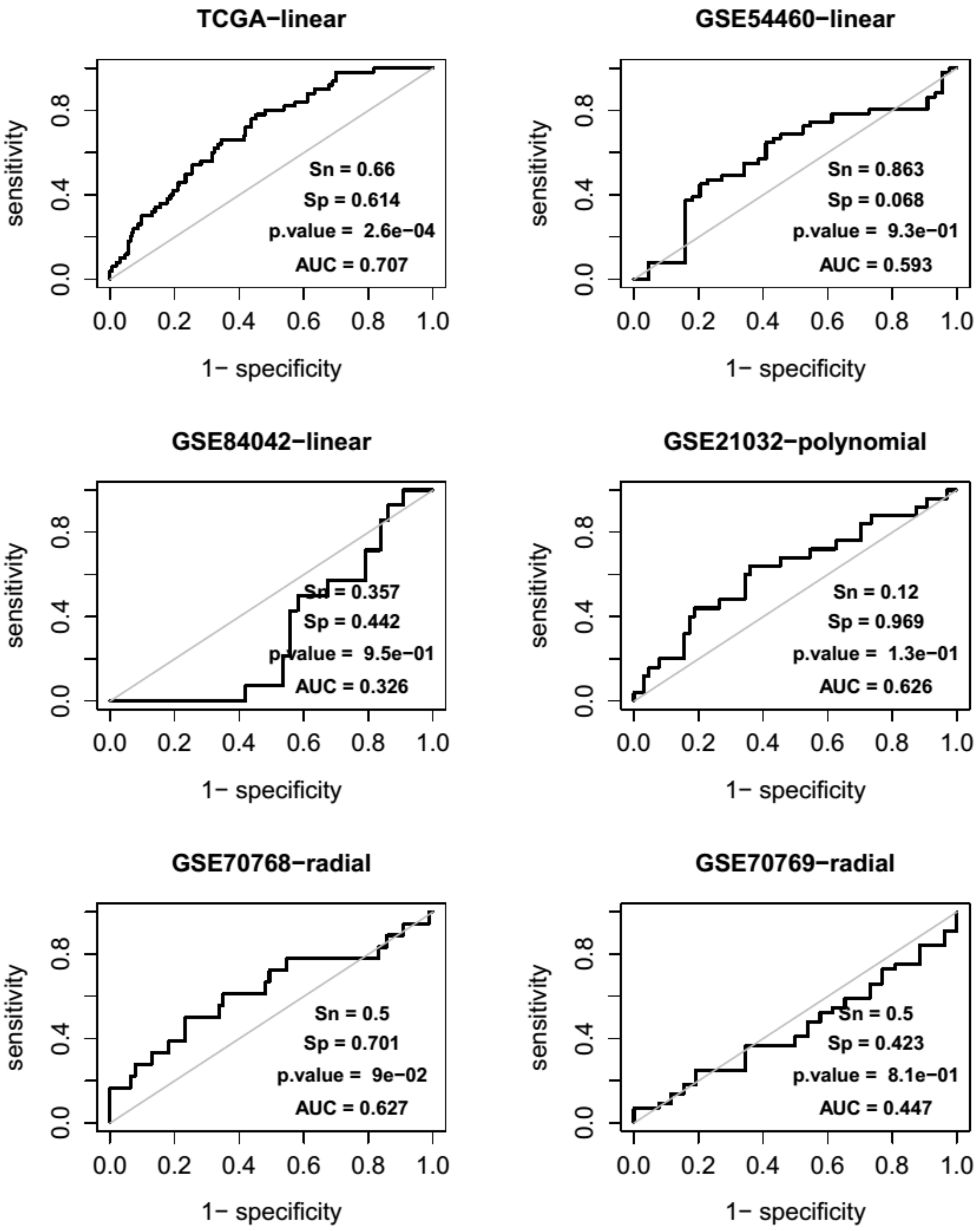

Supplementary Fig. S1: The performance of Wu's prognostic transcriptomic signature in the TCGA (-PRAD) dataset and five external datasets, i.e. the GSE54460 and others. The "-linear", "-polynomial" and "-radial" indicate the kernel functions used in the SVM models. The output BCR label (1 or -1) and numeric decision values (i.e. transcriptomic risk score (TRS), of a patient in the GSE70769 were predicted by the model trained using the GSE70768 dataset. For the patients in other cohorts, the labels and scores are predicted via LOOCV. Together with the actual BCR labels, the output BCR labels and TRSs were used to calculate a 2×2 contingency table for estimating the p-value and to generate the ROC curve, respectively. Sn and Sp denote sensitivity and specificity, respectively.

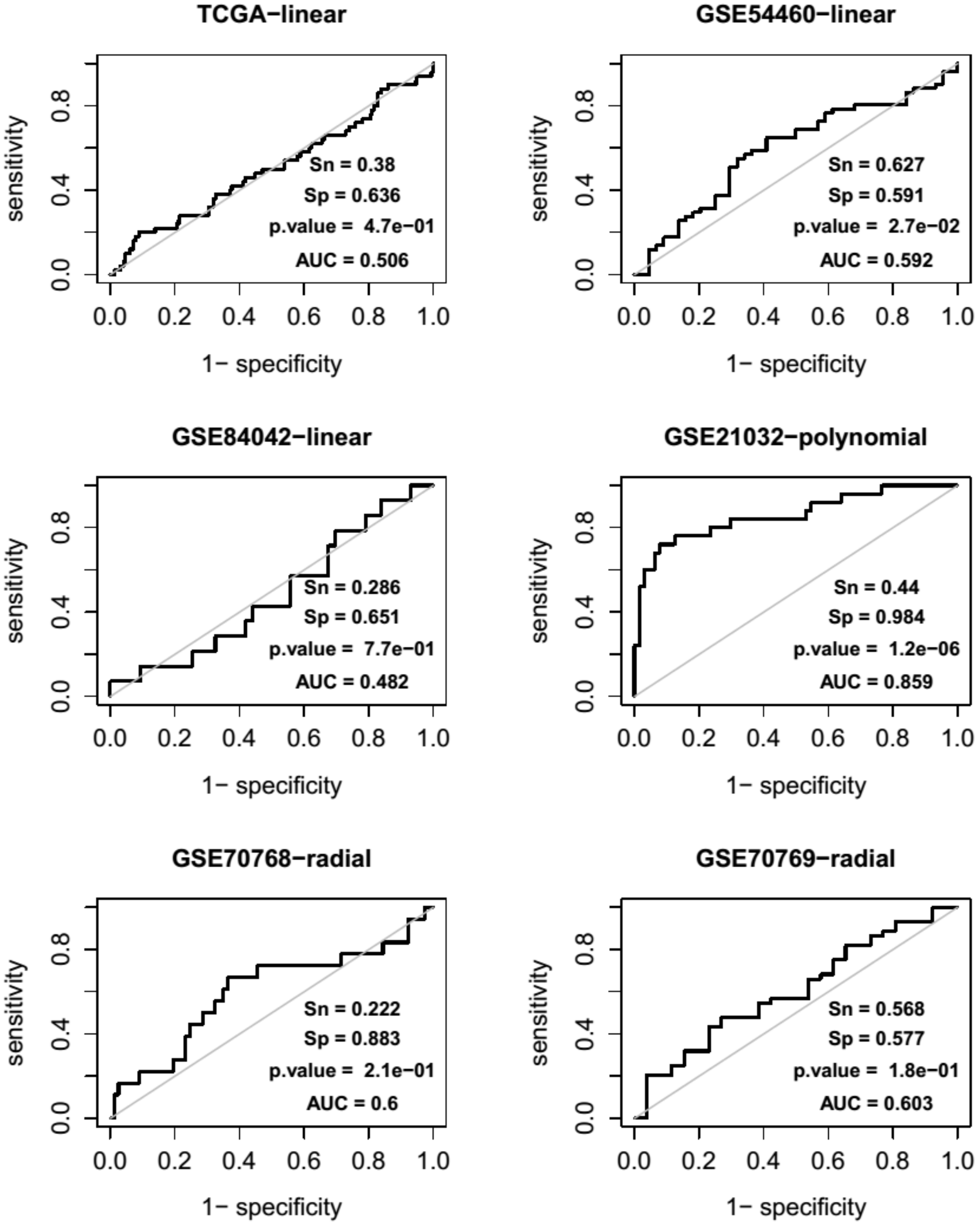

Supplementary Fig. S2: The performance of Li's prognostic transcriptomic signature in the TCGA (-PRAD) dataset and five external datasets, i.e. the GSE54460 and others. The "-linear", "-polynomial" and "-radial" indicate the kernel functions used in the SVM models. The output BCR label (1 or -1) and numeric decision values (i.e. transcriptomic risk score (TRS), of a patient in the GSE70769 were predicted by the model trained using the GSE70768 dataset. For the patients in other cohorts, the labels and scores were predicted via LOOCV. Together with the actual BCR labels, the output BCR labels and TRSs were used to calculate a 2×2 contingency table for estimating the p-value and to generate the ROC curve, respectively. Sn and Sp denote sensitivity and specificity, respectively.

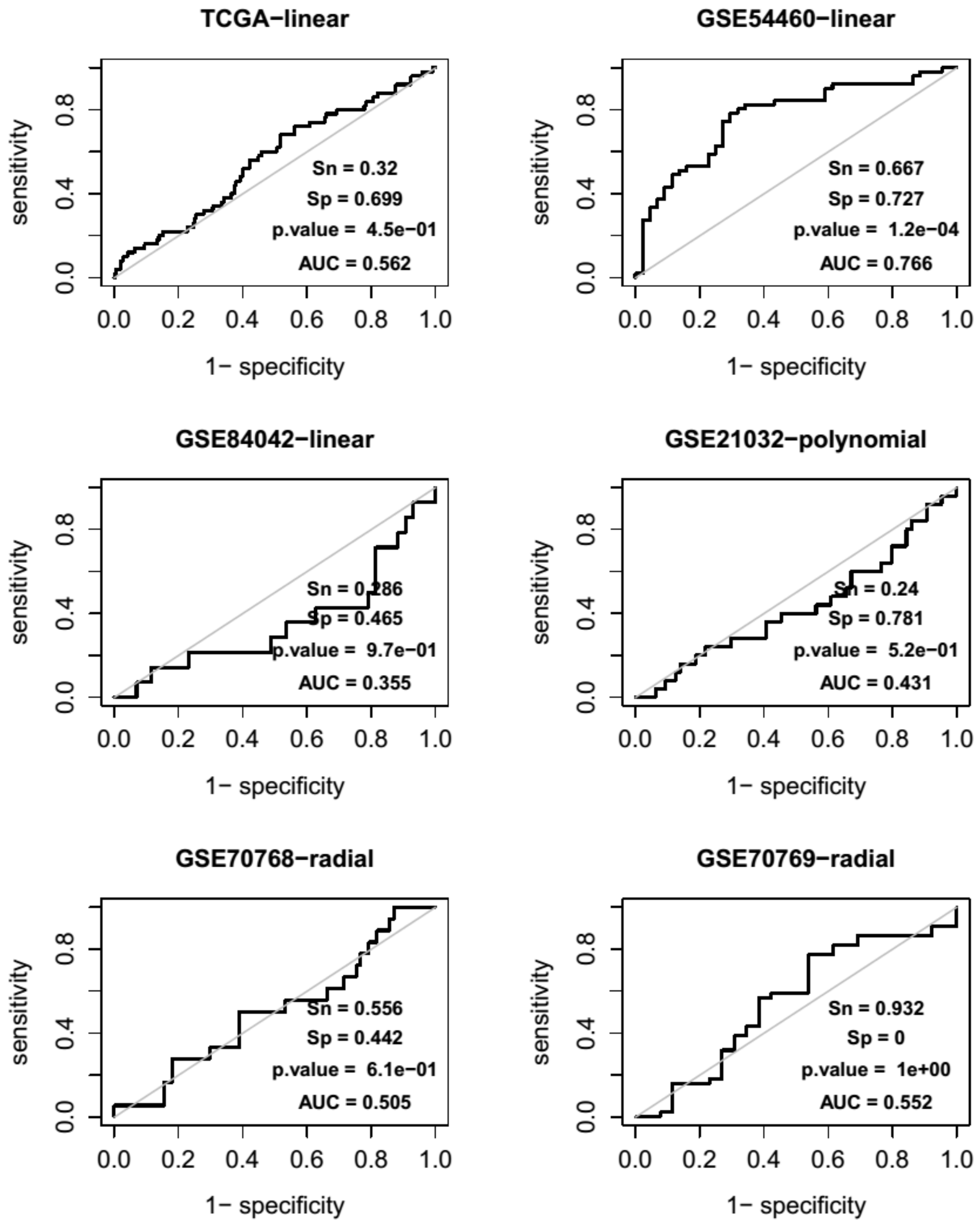

Supplementary Fig. S3 : The performance of Komisarof's prognostic transcriptomic signature in the TCGA (-PRAD) dataset and five external datasets, i.e. the GSE54460 and others. The "-linear", "-polynomial" and "-radial" indicate the kernel functions used in the SVM models. The output BCR label (1 or -1) and numeric decision values (i.e. transcriptomic risk score (TRS), of a patient in the GSE70769 were predicted by the model trained using the GSE70768 dataset. For the patients in other cohorts, the labels and scores are predicted via LOOCV. Together with the actual BCR labels, the output BCR labels and TRSs were used to calculate a 2×2 contingency table for estimating the p-value and to generate the ROC curve, respectively. Sn and Sp denote sensitivity and specificity, respectively.

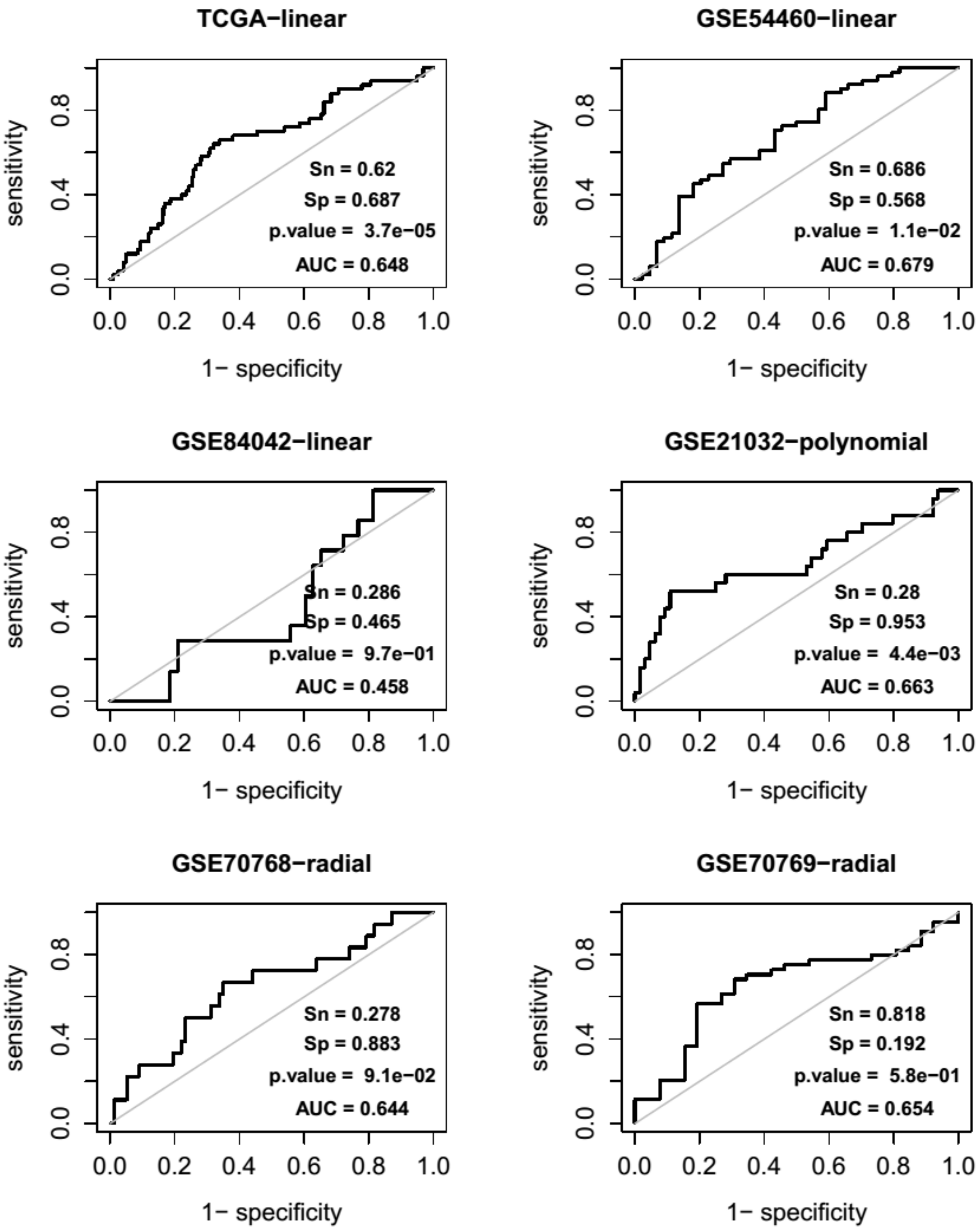

Supplementary Fig. S4: The performance of Erho's transcriptomic signature for BCR prediction in the TCGA (-PRAD) dataset and five external datasets, i.e. the GSE54460 and others. The "-linear", "-polynomial" and "-radial" indicate the kernel functions used in the SVM models. The output BCR label (1 or -1) and numeric decision values (i.e. transcriptomic risk score (TRS), of a patient in the GSE70769 were predicted by the model trained using the GSE70768 dataset. For the patients in other cohorts, the labels and scores are predicted via LOOCV. Together with the actual BCR labels, the output BCR labels and TRSs were used to calculate a 2×2 contingency table for estimating the p-value and to generate the ROC curve, respectively. Sn and Sp denote sensitivity and specificity, respectively.

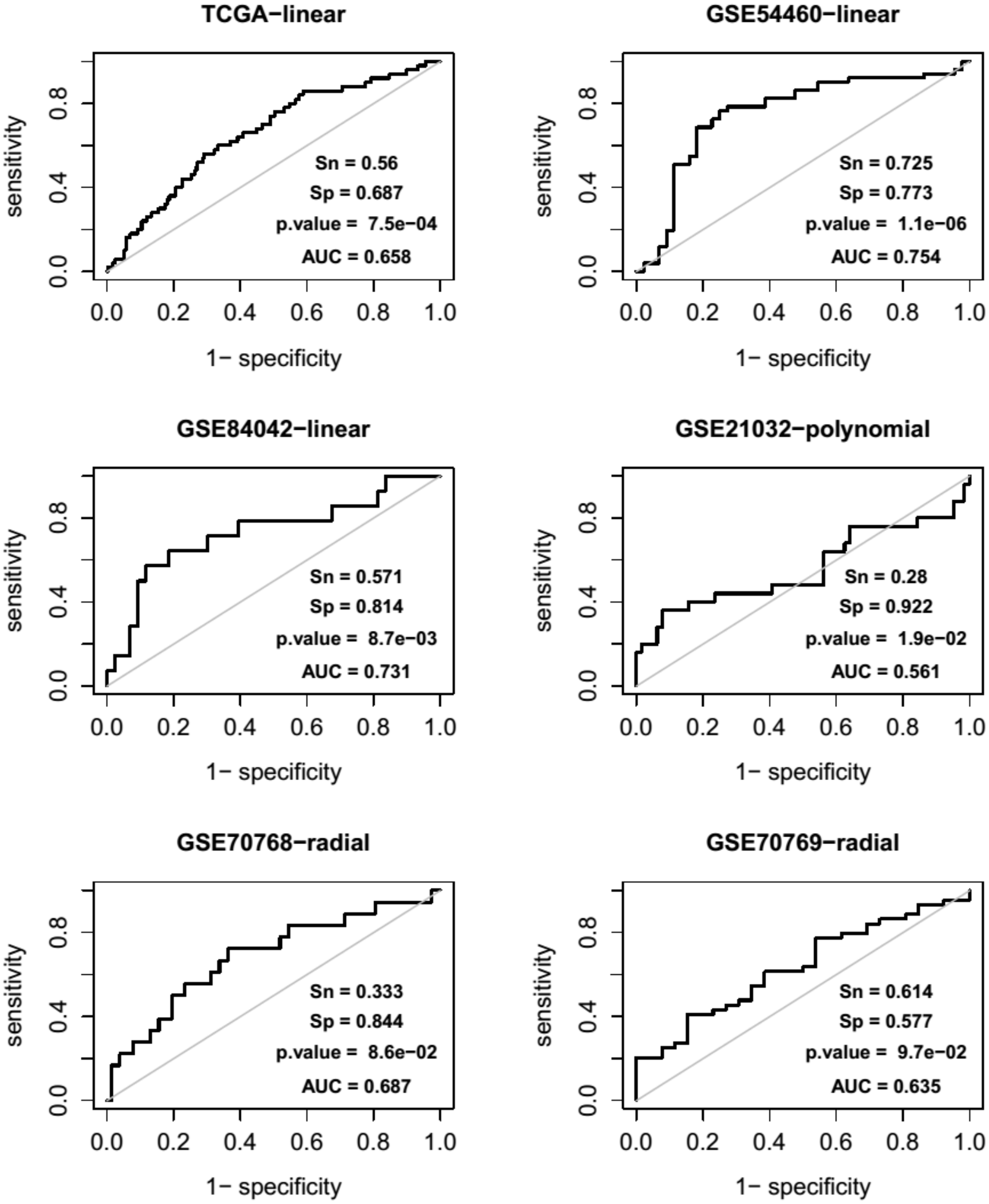

Supplementary Fig. S5: The performance of Knezevic-Klein's transcriptomic signature for BCR prediction in the TCGA (-PRAD) dataset and five external datasets, i.e. the GSE54460 and others. The "-linear", "-polynomial" and "-radial" indicate the kernel functions used in the SVM models. The output BCR label (1 or -1) and numeric decision values (i.e. transcriptomic risk score (TRS), of a patient in the GSE70769 were predicted by the model trained using the GSE70768 dataset. For the patients in other cohorts, the labels and scores are predicted via LOOCV. Together with the actual BCR labels, the output BCR labels and TRSs were used to calculate a 2×2 contingency table for estimating the p-value and to generate the ROC curve, respectively. Sn and Sp denote sensitivity and specificity, respectively.

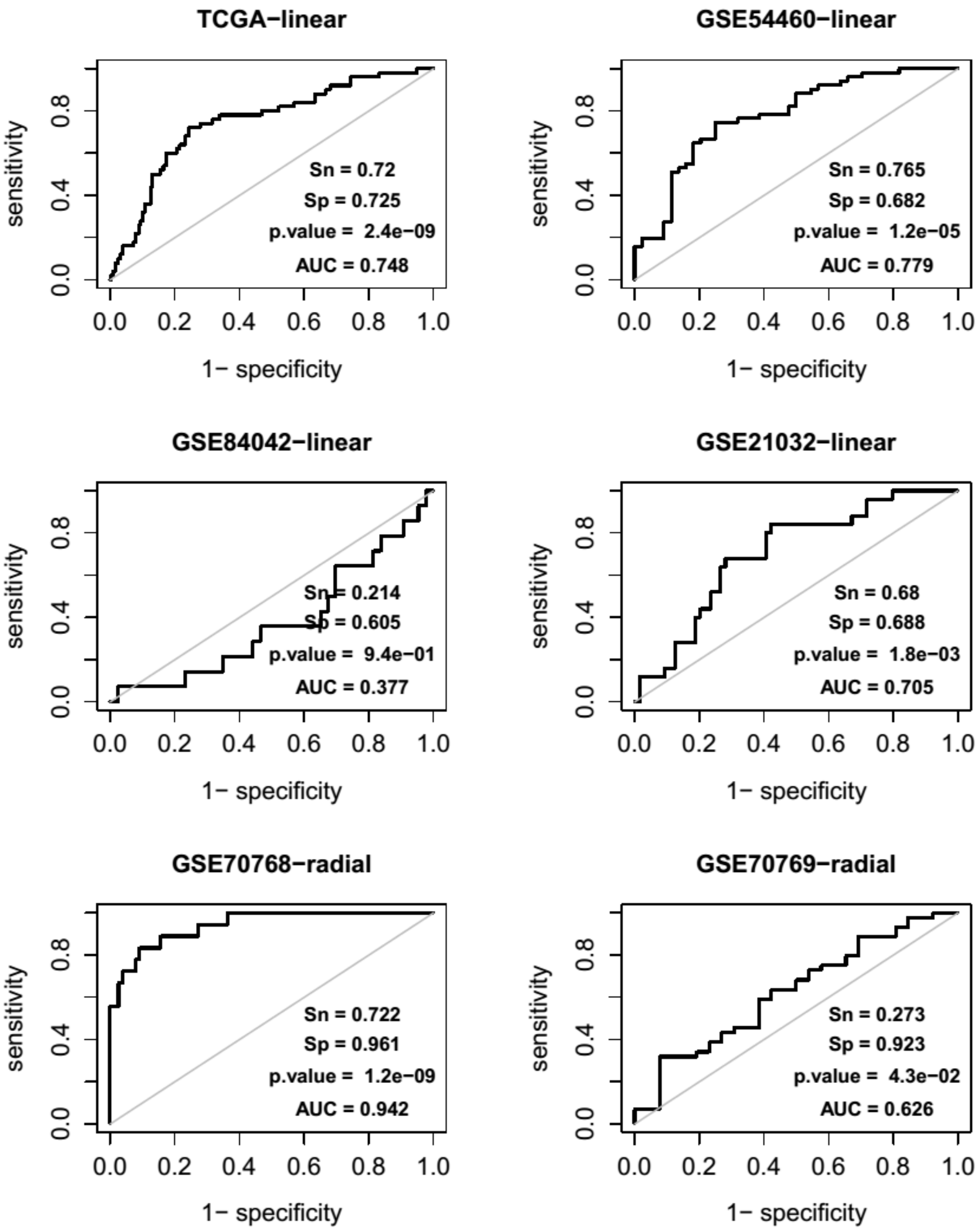

Supplementary Fig. S6: The performance of the immune-related prognostic transcriptomic signature for BCR prediction in the TCGA (-PRAD) dataset and five external datasets, i.e. the GSE54460 and others. The "-linear", "-polynomial" and "-radial" indicate the kernel functions used in the SVM models. The output BCR label (1 or -1) and numeric decision values, i.e. transcriptomic risk score (TRS) of a patient in the GSE70769 were predicted by the model trained using the GSE70768 dataset. For the patients in other cohorts, the labels and scores are predicted via LOOCV. Together with the actual BCR labels, the output BCR labels and TRSs were used to calculate a 2×2 contingency table for estimating the p-value and to generate the ROC curve, respectively. Sn and Sp denote sensitivity and specificity, respectively.

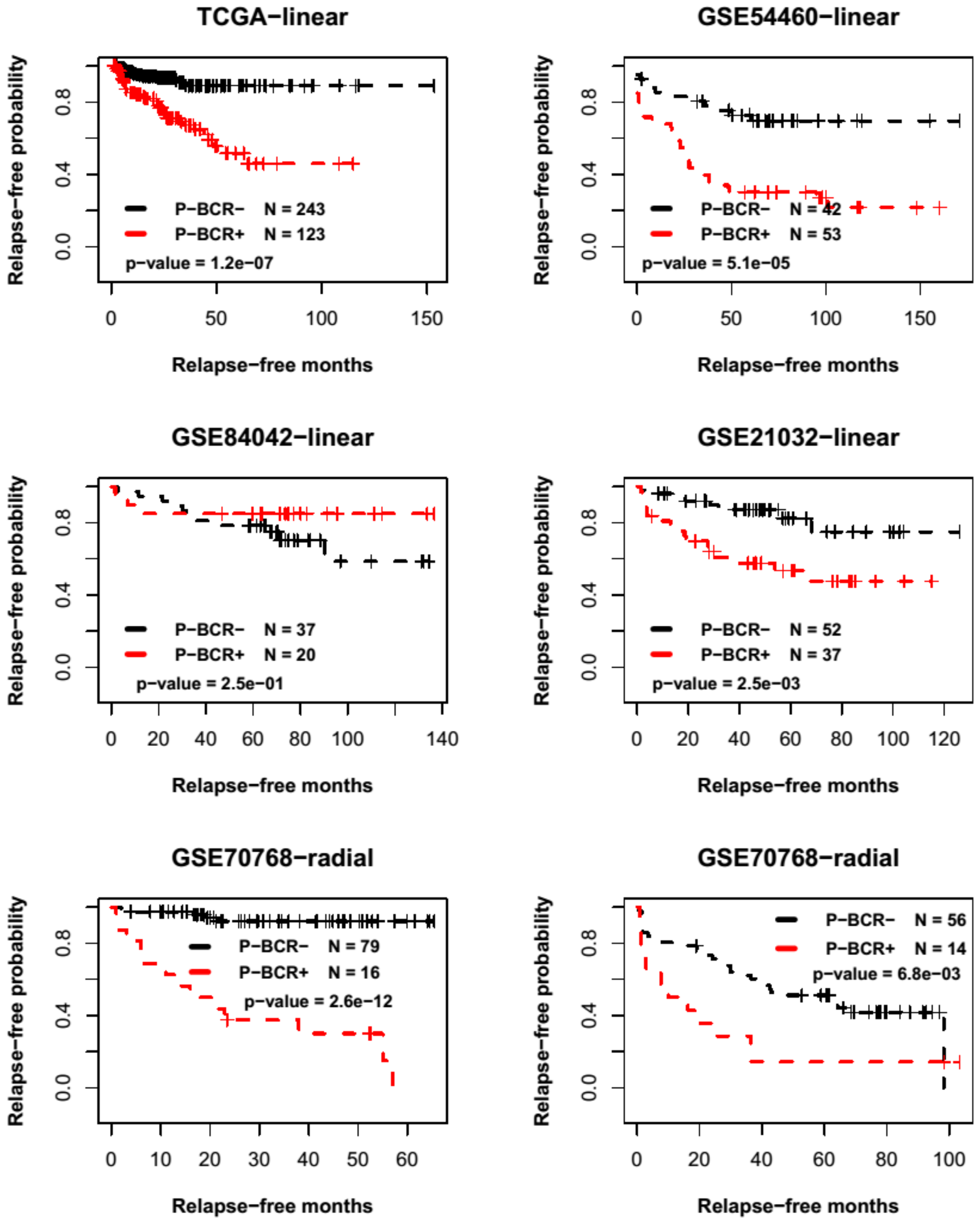

Supplementary Fig. S7: The association between RFS stratification and the BCR partition predicted using the immune-related prognostic transcriptomic signature, in the TCGA (-PRAD) dataset and five external datasets, i.e. the GSE54460 and others. The output BCR label (pre-BCR$^+$ and pre-BCR$^-$) of a patient in GSE70769 was predicted by the model trained using the GSE70768 dataset. For the patients in other cohorts, the labels were predicted via LOOCV. The survival profiles of pre-BCR$^+$ and pre-BCR$^-$ samples were depicted by red and black Kaplan Meier curves, respectively.

Reference

1 Wu, X. *et al.* A 10-gene signature as a predictor of biochemical recurrence after radical prostatectomy in patients with prostate cancer and a Gleason score ≥7. *Oncol Lett* **20**, 2906-2918, doi:10.3892/ol.2020.11830 (2020).

2 Li, X. *et al.* A qualitative transcriptional signature for predicting the biochemical recurrence risk of prostate cancer patients after radical prostatectomy. *Prostate* **80**, 376-387, doi:10.1002/pros.23952 (2020).

3 Komisarof, J. *et al.* A four gene signature predictive of recurrent prostate cancer. *Oncotarget* **8**, 3430-3440, doi:10.18632/oncotarget.13837 (2017).

4 McMurray, H. R. *et al.* Synergistic response to oncogenic mutations defines gene class critical to cancer phenotype. *Nature* **453**, 1112-1116, doi:10.1038/nature06973 (2008).

5 Erho, N. *et al.* Discovery and validation of a prostate cancer genomic classifier that predicts early metastasis following radical prostatectomy. *PLoS One* **8**, e66855, doi:10.1371/journal.pone.0066855 (2013).

6 Klein, E. A. *et al.* A 17-gene assay to predict prostate cancer aggressiveness in the context of Gleason grade heterogeneity, tumor multifocality, and biopsy undersampling. *Eur Urol* **66**, 550-560, doi:10.1016/j.eururo.2014.05.004 (2014).

7 Knezevic, D. *et al.* Analytical validation of the Oncotype DX prostate cancer assay - a clinical RT-PCR assay optimized for prostate needle biopsies. *BMC Genomics* **14**, 690, doi:10.1186/1471-2164-14-690 (2013).
